# Reverse Remodeling in Human Heart Failure after Cardiac Resynchronization Therapy Is Associated With Reduced RHO-Kinase Activation

**DOI:** 10.3389/fphar.2021.565724

**Published:** 2021-04-23

**Authors:** Maria Paz Ocaranza, Jorge E. Jalil, Rodrigo Altamirano, Ana de León, Jackeline Moya, Alejandra Lonis, Luigi Gabrielli, Paul Mac Nab, Samuel Córdova, Alejandro Paredes, Ismael Vergara, Alex Bittner, Karime Sabat, Karla Pastorini

**Affiliations:** ^1^ Pontificia Universidad Católica de Chile, School of Medicine, Department of Cardiovascular Diseases, Santiago, Chile; ^2^ Center for New Drugs for Hypertension (CENDHY), Pontificia Universidad Católica de Chile, Santiago, Chile; ^3^ Pontificia Universidad Católica de Chile, School of Medicine, Advanced Center for Chronic Diseases (ACCDiS), Santiago, Chile; ^4^ Servicio de Cardiología, Hospital Sótero del Río, Santiago, Chile

**Keywords:** Rho kinase, heart failure, remodeling, reverse remodeling, resynchronization, Mypt, HFrEF

## Abstract

**Background:** Reverse remodeling is a clinically relevant endpoint in heart failure with reduced ejection fraction (HFrEF). Rho-kinase (ROCK) signaling cascade activation correlates with cardiac remodeling and left ventricular (LV) systolic dysfunction in HFrEF patients. Cardiac resynchronization therapy (CRT) is effective in HFrEF, especially when there is a left bundle block, as this treatment may stimulate reverse remodeling, thereby improving quality of life and prolonging survival for patients with this severe condition. Here, we evaluate the hypothesis that ROCK activation is reduced after effective CRT in HFrEF.

**Methods:** ROCK activation in circulating leukocytes was evaluated in 28 HFrHF patients, using Western blot (myosin light chain phosphatase subunit 1 phosphorylation, MYPT1p/t), before and three months after initiation of CRT. LV systolic function and remodeling were assessed by echocardiography.

**Results:** Three months after CRT, LV ejection fraction increased an average of 14.5% (*p* < 0.001) in 13 patients (responders), while no change was observed in 15 patients (non-responders). End-systolic diameter decreased 16% (*p* < 0.001) in responders, with no change in non-responders. ROCK activation in PBMCs decreased 66% in responders (*p* < 0.05) but increased 10% in non-responders (NS). LV end-diastolic diameter was also 5.2 mm larger in non-responders vs. responders (*p* = 0.058). LV ejection fraction, systolic diameter, and ROCK activation levels were similar in both groups at baseline.

**Conclusion:** In HFrEF patients, 3 months of effective CRT induced reverse myocardial remodeling, and ROCK activation was significantly decreased in circulating leukocytes. Thus, decreased ROCK activation in circulating leukocytes may reflect reverse cardiac remodeling in patients with heart failure.

## Introduction

In patients with heart failure and low or reduced ejection fraction, reverse myocardial remodeling or reverse remodeling is generally described as increased left ventricular ejection fraction (LVEF) and reduced LV diameter or volume (Choi et al., 2013; Maki and Takeda, 2020). Reverse cardiac remodeling occurs in approximately 30% of patients with nonischemic dilated cardiomyopathy (NIDCM) (Choi et al., 2013). In dilated cardiomyopathy with left ventricular systolic dysfunction, clinical deterioration and prognosis depend mainly on the disease severity and the presence of reverse myocardial or cardiac remodeling (Reichart et al., 2019).

LV reverse remodeling correlates with long-term prognosis in patients hospitalized with decompensated HFrEF and LVEF <40% at hospital discharge (Abe et al., 2020; Maki and Takeda, 2020). After 4 months of follow-up, LVEF normalizes or recovers to mid-range in nearly 60% of patients. These changes are associated with significantly lower cardiac event rates and all-cause mortality and correlate with younger age, absence of atrial fibrillation, smaller LV diastolic diameter, and higher LVEF at first discharge (Abe et al., 2020). In previous clinical trials of HFrEF, patients receiving beta-blockers, angiotensin converting enzyme inhibitors (ACEIs), angiotensin II type 1 receptor blockers (ARBs), or ivabradine, LVEF has improved by 2–12% over the course of 5–20 months (Tardif et al., 2011; Sze and Daubert, 2018; Maki and Takeda, 2020).

Cardiac resynchronization therapy has been shown to be very effective in promoting reverse remodeling. Furthermore, its clinical efficacy has been specifically associated with LV reverse remodeling. In the Multicenter Automatic Defibrillator Implantation Trial-CRT (MADIT-CRT), left bundle branch block and non-ischemic etiology of cardiomyopathy were significant predictors of super-responders to CRT (25% of the total 752 patients showed LVEF improvements ≥14.5% twelve months after CRT implantation) (Hsu et al., 2012; Sze and Daubert, 2018). Baseline echocardiographic parameters, including LVEF >30%, LV end-systolic volume <170 ml, and left atrial volume index ≤45 ml/m^2^ predicted LVEF normalization after CRT implantation (Ruwald et al., 2014).

While LV reverse remodeling is critically important in HF management, the factors that predict reverse remodeling, as well as the underlying molecular processes, remain to be elucidated (Maki and Takeda, 2020).

In HFrEF, activation of the renin-angiotensin aldosterone and sympathetic systems significantly contributes to pathological cardiac remodeling and disease worsening (Jalil et al., 2005; Shimokawa et al., 2016). Both noradrenalin and angiotensin II activate the small protein RhoA signaling pathway and its target Rho-associated coiled-coil containing protein kinase, or Rho-kinase (ROCK). Activated ROCK phosphorylates intracellular proteins that promote cardiac hypertrophy, LV ventricular dysfunction, fibrosis, inflammation, and apoptosis (Loirand and Pacaud, 2014; Shimokawa et al., 2016; Hartmann et al., 2015; Surma et al., 2011).

Experimental HErEF models have been used to explore the role of ROCK as a molecular mechanism regulating cardiac function and reverse remodeling. In the preclinical model of congestive heart failure in the Dahl hypertensive rat, the specific ROCK inhibitor Y-27632, administered from the left ventricular hypertrophic phase to the overt heart failure phase, myocardial contractility was reduced by 47% and it was significantly ameliorated by Y-27632 (Kobayashi et al., 2002). Moreover, LV end-diastolic pressure was significantly increased in the CHF than control animals (20.3 vs. 4.9 mm Hg) but it was significantly reduced by ROCK inhibition to 9.9 mm Hg. This finding is consistent with the relevance of the ROCK pathway to the pathogenesis of myocardial dysfunction and cardiovascular remodeling (Kobayashi et al., 2002). Another study investigated the molecular mechanisms by which ROCK activation in the myocardium provokes remodeling and decreased cardiac systolic function after myocardial infarction (MI). These authors assessed myocardial hypertrophy, fibrosis, and systolic LV function in rats receiving the ROCK inhibitor fasudil (Mera et al., 2016). After MI, deterioration in LV systolic function was significantly associated with cardiac ROCK activation. Phosphorylation of downstream targets of ROCK that promote ventricular remodeling, such as ERK/GATA-4 and β-MHC pathway proteins, was thought to be responsible for these effects. ROCK inhibition with fasudil significantly enhanced systolic function, reduced myocardial fibrosis, and normalized β-MHC and ERK/GATA-4 phosphorylation levels (Mera et al., 2016).

In cardiac failure patients with low or reduced ejection fraction (HFrEF), ROCK activation in peripheral blood mononuclear cells (PBMCs) is markedly elevated (Ocaranza et al., 2011; Dong et al., 2012; Do e et al., 2013) and inversely correlated with ejection fraction (Ocaranza et al., 2011). Furthermore, the Rho-kinase pathway is activated in circulating leukocytes from patients with HFrEF under optimal pharmacological treatment. Rho-kinase pathway activation is directly associated with apoptosis and higher levels of circulating neurohormones in this population (Ocaranza et al., 2020). Nevertheless, there are no clinical studies in HFrEF assessing the relationship between ROCK activation and reverse remodeling.

The present study assesses the role of ROCK activation in reverse cardiac remodeling in human HFrEF by comparing ROCK activation levels in PBMCs from patients with and without reverse remodeling 3 months after cardiac resynchronization therapy.

## Methods

### Study Design

This prospective clinical study included 28 consecutive patients with chronic HFrEF and left bundle branch block referred for CRT by their cardiologists. Patients were also evaluated by a cardiologist specializing in heart failure prior to study enrollment.

This clinical study complied with the principles of the Helsinki Declaration and was authorized by the Human Research Ethics Committee (Pontificia Universidad Católica de Chile School of Medicine). All participants signed an informed consent form prior to any study procedures.

Twenty-eight sequential HFrEF patients with an ejection fraction ≤35% were evaluated. All patients were receiving optimal pharmacological treatment according to the 2012 American College of Cardiology, American Heart Association, and Heart Rhythm Society CRT guidelines. The group included patients with asymptomatic and mildly symptomatic HF (Epstein et al., 2013). All patients were in sinus rhythm.

Exclusion criteria were myocardial infarction within the previous 6 months, clinical indication for coronary artery revascularization, cancer in the last 4 years, active infection within the previous 8 weeks, use of high-dose statins, and other major chronic illness such as renal, respiratory, or hepatic failure.

CRT implantations were performed by electrophysiologists using conventional devices, cardiac electrodes, and procedures. One electrode was positioned in the right ventricle and the other in the coronary sinus. No study patients experienced clinical complications during or after the procedure.

### Blood Samples, Electrocardiogram, and 6 min Walk Test

Venous blood samples were obtained before and 3 months after CRT for standard clinical laboratory measurements and to obtain PBMC samples to determine ROCK activation as previously reported (Ocaranza et al., 2011; Gabrielli et al., 2014; Ocaranza et al., 2020). Twelve-lead electrocardiograms were also obtained at this timepoint, and QRS width was measured.

Additionally, in order to assess functional capacity immediately before and 3 months after initiation of CRT, a 6 min walk test was performed in a flat 27-m corridor. Heart rate and oxygen saturation were measured with a pulse oximeter. The Borg Breathlessness Scale was used to assess patient-reported dyspnea during physical activity.

### Cardiac Dimensions and Functional Echocardiographic Measurements

Echocardiographic measurements were performed using a Philips iE33 instrument with a 2.5-MHz transducer (Andover, MA) to evaluate cardiac size and LV ejection fraction at the time of blood sampling, immediately before and 3 months after the CRT procedure, as previously described (Ocaranza et al., 2011; Gabrielli et al., 2014; Ocaranza et al., 2020). Blind echocardiographic studies were performed following American Society of Echocardiography clinical guidelines (Lang et al., 2005).

### Protein Extracts for Western Blots Obtained From Circulating Leukocytes

PBMCs were isolated from five volumes of blood with ethylenediaminetetraacetic acid and poured over 5 volumes of density gradient cell separation medium, as previously described (Ocaranza et al., 2011; Gabrielli et al., 2014). Leukocytes were separated, resuspended, and placed in lysis buffer with sodium chloride, the detergent lysis buffer NP-40, Tris dodecyl sulfate, and deoxycholate, as well as leupeptin, aprotinin, and phenylmethylsulfonyl fluoride. Lowry reagent was used for protein determination, and ROCK activation was assessed by Western blotting as described previously (Ocaranza et al., 2011; Gabrielli et al., 2014).

### Western Blot Analysis

Fractions of soluble protein were heated (95°C) for 5 min with sodium dodecyl sulfate (SDS) polyacrylamide, as previously reported (Ocaranza et al., 2011; Gabrielli et al., 2014). Identical quantities of protein were loaded, separated, and transferred into a nitrocellulose membrane (400 mA for 2 h on ice). Bovine serum albumin was used for blocking (room temperature). Blots were incubated overnight with the primary antibody (4°C). The amount of protein was gauged by chemiluminescence using the ECL-Plus kit (Perkin Elmer), which contains horseradish peroxidase (HRP) substrate. The Syngene G-Box system was used to obtain digital images, and densitometric analysis was performed with UN-SCAN-IT™ software from Silk Scientific Corporation.

Blots with both antibodies, anti-MYPT1 (1/500, Cell signaling, Cat 2,634), and anti-*p*-MYPT1 (1/500, CycLex, Cat CY-P1025) were incubated overnight (Ocaranza et al., 2011; Gabrielli et al., 2014). Afterward, the blots were washed and incubated with a secondary HRP-labeled antibody (goat anti-rabbit IgG, 1/7,500, Thermo Fisher Scientific, Cat 31,466 or goat anti-mouse IgG, 1:10,000, Santa Cruz, Cat sc 2005) for 2 h β-actin (1/10,000, Sigma, Cat A2228) was used as a protein loading standard.

### Assessment of Apoptosis by DNA Fragmentation

2 × 10^4^ PBMC cells were collected and washed with phosphate buffered saline (PBS). The cells were then fixed with 4% paraformaldehyde within a 1 cm^2^ area and air-dried for 24 h. Cells were washed twice (PBS). Cells were incubated with permeabilization solution for 15 min, the solution removed, and TUNEL analysis performed using the *In Situ* Cell Death Detection Kit, POD (Roche Inc. Indianapolis, United States). Only cells showing TUNEL-positive nuclei plus chromatin margination were considered apoptotic. 400 consecutive cells (within 20 sequential fields) were analyzed (40x). We compared the number of total nuclei and apoptotic nuclei to calculate the percentage of apoptotic cells (Ocaranza et al., 2020).

### Statistical Analysis

According to our previous measurements of ROCK activation in circulating leukocytes measured as MYPT1 phosphorylation (the ratio between phosphorylated and total MYPT1, or MYPT1-p/t) in HFrEF patients, the sample size was calculated assuming a reduction of MYPT1-p/t in circulating leukocytes by one standard deviation after effective reverse remodeling 3 months after RCT (power 80%, alfa error 1%, non responders patients rate 25% and lost to follow up rate = 15%). Results are shown here as mean ± SD. In order for testing for normality we used both the Kolmogorov-Smirnov as well as the Shapiro-Wilk tests. Baseline differences between responders and non-responders were analyzed using a *t*-test (or the Mann Whitney *U* test when data were not normally distributed). A Student paired *t*-test was used to compare changes within each group 3 months after CRT when data were normally distributed (or the Wilcoxon Signed Rank Test when data were not normally distributed). A *p*-value ≤ 0.05 was considered statistically significant.

## Results

### Clinical Characteristics, Laboratory Tests, and Exercise Capacity

This study included 28 HFrEF patients (mean age 60.1 years, body mass index 28.5 kg/m^2^, heart rate 68.6 bpm, disease duration according to symptoms 40.6 months). Of the total sample, 67.9% were male, 53.6% were in NYHA functional class II HF, and all had a left bundle branch block.

The etiology of HFrEF in the patients was mainly dilated cardiomyopathy treated with standard pharmacological therapy. Of the total sample, 10 (35.7%), 14 (50%), 22 (78.6%), 23 (82.1%), 16 (57.1%), 13 (46.4%), and 26 (93%) patients were treated with ACE-Is, ARB, beta-lockers, diuretics, statins, aspirin, and spironolactone, respectively.

**FIGURE 1:**
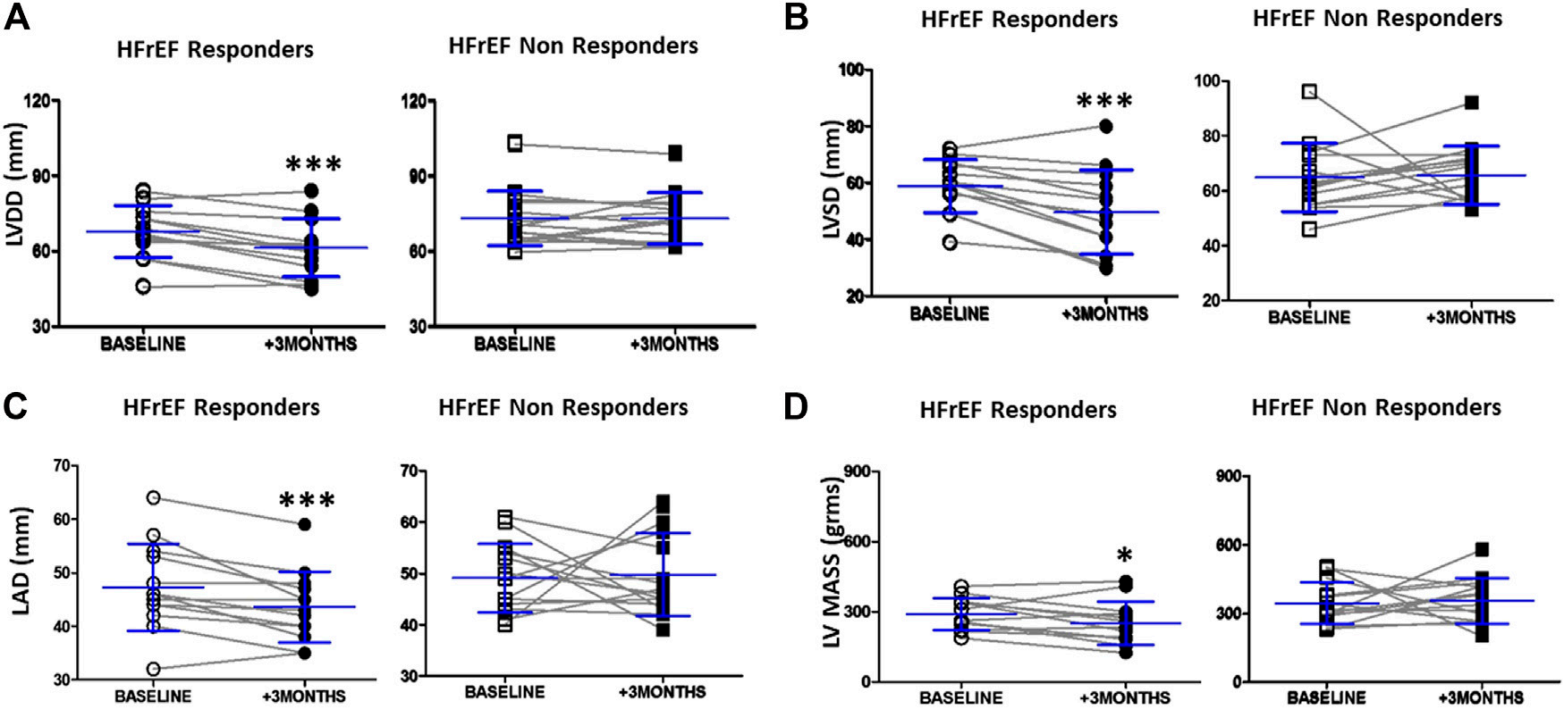
LV and left atrial dimensions before and after 3 months of cardiac resynchronization therapy in responders and non responders HFrEF patients **(A)** Dot graph of left ventricle diastolic diameter in responders patients (*n* = 13, white circles = baselines and black circles = + 3 months) and non responders patients (*n* = 15, white squares = baselines and black squares = + 3 months) **(B)** Dot graph of left ventricle systolic diameter in responders patients (*n* = 13, white circles = baselines and black circles = + 3 months) and non responders patients (*n* = 15, white squares = baselines and black squares = + 3 months) **(C)** Dot graph of left atrial diameter in responders patients (*n* = 13, white circles = baselines and black circles = + 3 months) and non responders patients (*n* = 15, white squares = baselines and black squares = + 3 months) **(D)** Dot graph of left ventricle mass in responders patients (*n* = 13, white circles = baselines and black circles = + 3 months) and non responders patients (*n* = 15, white squares = baselines and black squares = + 3 months). Data are shown as mean ± SD. Symbols: ***p = < 0.001; ** = *p*< 0.01 (non parametric Wilcoxon Signed Rank Test); * = *p*< 0.05 vs. baseline, respectively. Abbreviations: DD = end diastolic diameter, SD = end systolic diameter, LAD = left atrial diameter, LV = left ventricle.

**FIGURE 2:**
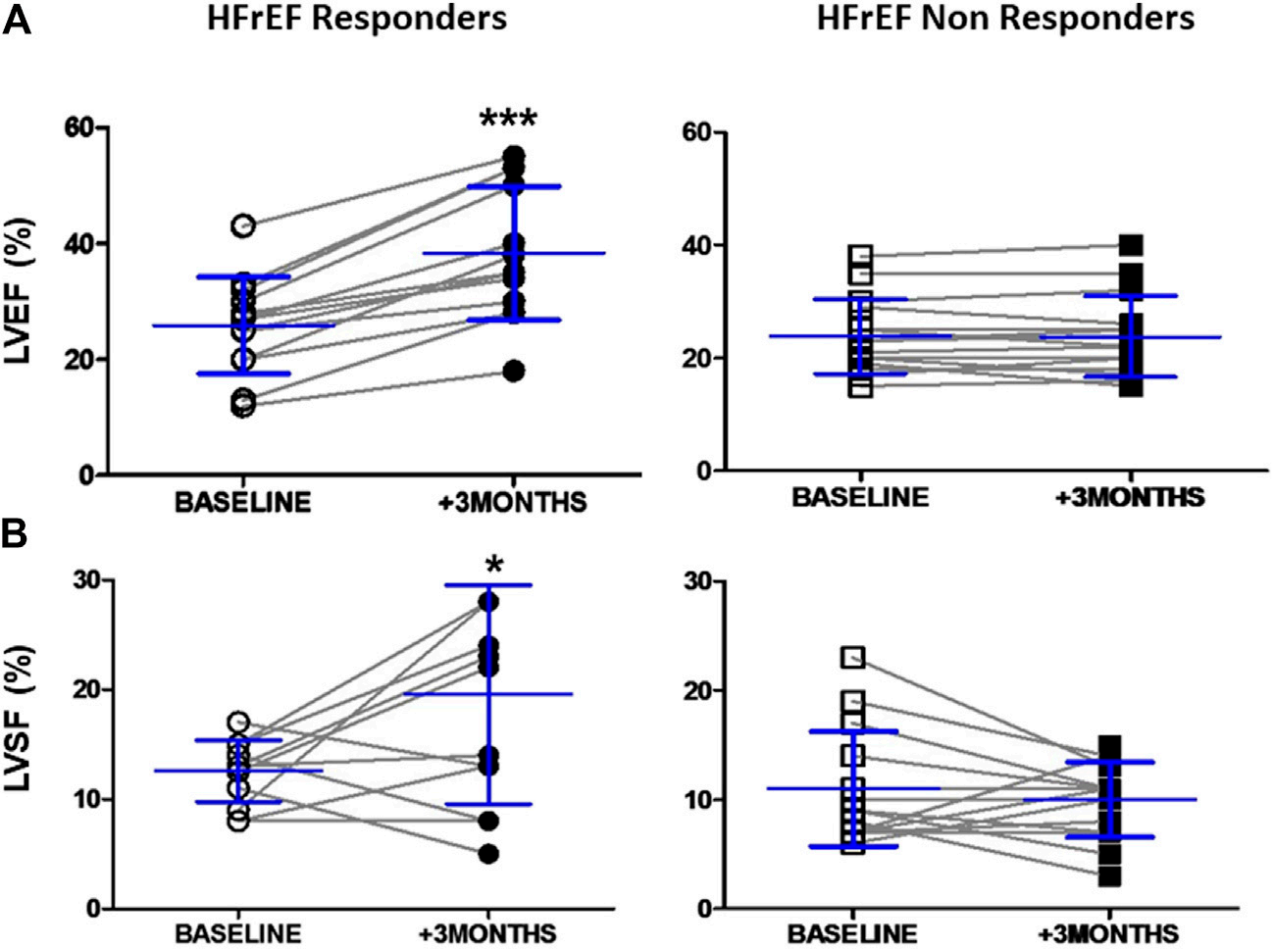
LV systolic function before and 3 months after cardiac resynchronization therapy in responders and non responders HFrEF patients **(A)** Dot graph of left ventricle eyection fraction in responders patients (*n* = 13, white circles = baselines and black circles = + 3 months) and non responders patients (*n* = 15, white squares = baselines and black squares = + 3 months) **(B)** Dot graph of shortening fraction in responders patients (*n* = 13, white circles = baselines and black circles = + 3 months) and non responders patients (*n* = 15, white squares = baselines and black squares = + 3 months). Data shown as mean ± SD. Symbols: *** = *p*< 0.001; * = *p*< 0.05 (non parametric Wilcoxon Signed Rank Test) vs. baseline, respectively. Abbreviations: EF = Ejection fraction, SF = Shortening fraction.

All patients underwent CRT device placement without clinical complications. Three months after CRT, LV ejection fraction improved in 13 patients (responders), with no change in LVEF in 15 (non-responders). Baseline clinical characteristics, laboratory tests, exercise capacity, and LV systolic function and dimensions were comparable in the two groups (Tables 1, 2; Figures 1, 2).

**TABLE 1:** Baseline functional class, demographics, heart rate, etiology, cardiac dimensions and LV function and pharmacological treatment in Responders and Non Responders HFrEF patients (*n* = 28).

	Responders (*n* = 13)	No responders (*n* = 15)
Age (y)	58.8 ± 8.6	61.1 ± 10.7
Men (%)	53.8	80.0
Weight (kg)	81.3 ± 12,6	75.7 ± 10.7
Body mass index (kg/m^2^)	30.2 ± 5.2	26.9 ± 3.4
Heart rate (bpm)	72.2 ± 11.3	65.3 ± 11.15
Evolution time by symptoms (months)	29.2 ± 31.6	50.5 ± 36.9
NYHA functional class		
I-II (%)	0	6.7
II (%)	61.5	46.7
II-III (%)	23.1	20
III (%)	15.4	26.7
Etiology		
Idiopathic dilated cardiomyopathy (%)	100	86.7
Coronary heart disease (%)	7.7	6.7
Hypertensive (%)	7.7	0
Aortic valve surgery (%)	0	13.3
Hypertensive cardiomyopathy (%)	0	3.6
Cardiac dimensions and LV function (echo)		
LV end systolic diameter (mm)	59.1 ± 9.3	64.8 ± 12.4
LV end diastolic diameter (mm)	67.5 ± 10.4	72.7 ± 10.8
Left atrial diameter (mm)	47.3 ± 8.1	49.2 ± 6.7
LV ejection fraction (%)	25.8 ± 8.3	23.9 ± 6.6
LV shortening (%)	12 ± 3	11 ± 5
Pharmacological treatment		
ACE inhibitors (%)	53.8	20.0
Angiotensin receptor blockers (%)	15.4	100
Mineralocorticoid receptor blockers (%)	100	80
Sacubitril/Valsartán (%)	7.7	0
Betablockers (%)	84.6	73.3
Diuretics (%)	84.6	80
Statins (%)	53.8	60
Aspirine (%)	53.8	40
Antiarrhythmic agent (%)	7.7	33.3
Digoxine (%)	7.7	20
Metformine (%)	7.7	26.7
Anticoagulants (%)	7.7	20.0

Values shown as mean ± SD.

**TABLE 2:** Blood pressure, heart rate, blood chemistry and exercise capacity 3 months after cardiac resynchronization therapy.

	Responders (*n* = 13)		Non responders (*n* = 15)	
	Baseline	3 months after CRT	Baseline	3 months after CRT
QRS width (ms)	170.6 ± 19.8	132.1 ± 32.68**	165.9 ± 22	135.4 ± 34.3**
Heart rate (bpm)	72.2 ± 11.3	68 ± 8.1*	65.3 ± 11.1	70.5 ± 9.2
Creatinine (mg/dl)	1 ± 0.1	0.9 ± 0.1*	1 ± 0.2	1.1 ± 0.3
Hematocrit (%)	41.5 ± 3.7	40.2 ± 4.3	43.1 ± 4.7	42.3 ± 3.8
WBC (x10^3^ cells/mL)	7.8 ± 3	7 ± 2.2	6.7 ± 1.5	6.4 ± 1.3
SGOT (U/L)	24.6 ± 7.1	23.6 ± 5.4	25.5 ± 9.5	26.5 ± 9.5
Potassium (mmol/L)	4.7 ± 0.5	4.5 ± 0.5	4.6 ± 0.5	4.5 ± 0.4
Distance 6 min (m)	346.5 ± 112.4	454.2 ± 49.2**	420.8 ± 106.4	496.2 ± 100.3**
Borg 6 min	5.7 ± 2.7	2.5 ± 1.4***	4.6 ± 2	3.1 ± 1.6*
Heart rate 6 min (bpm)	82 ± 13.6	76 ± 10.3	75.8 ± 13.3	78.1 ± 11.8
SBP 6 min (mm hg)	118.3 ± 20.3	124.5 ± 21.8	123.1 ± 19.3	127.1 ± 26.8
DBP 6 min (mm hg)	70.3 ± 12.3	68 ± 8.2	65.9 ± 9	70.5 ± 11
0_2_ saturation 6 min (%)	97.7 ± 1.5	97.5 ± 1.5	96.2 ± 3.1	96 ± 2.5

Values shown as mean ± SD. Symbol: ***p < 0.001; **p < 0.01 (non parametric Wilcoxon Signed Rank Test) and *p < 0.05 vs baseline. WBC = white blood cells; SGOT = serum glutamic oxaloacetic transaminase; SBP = systolic blood pressure; DBP = diastolic blood pressure.

Three months after CRT, both groups showed a similar and significant narrowing of the QRS and a significant increase in functional capacity as assessed by distance walked during the 6 min walk test. Perceived dyspnea during exercise was also significantly reduced in both groups as measured by the Borg scale at 6 min. Baseline heart rate was reduced in responders but not in non-responders (Table 2).

### Reverse Remodeling 3 months After CRT Assessed by Echocardiography

Three months after device implantation, end-diastolic LV diameter, end-systolic LV diameter, and left atrial diameter were significantly smaller in CRT responders, with reductions of 8, 16, and 7.8% respectively (*p* < 0.001, Figure 1). LV mass was also reduced in responders (*p* < 0.05, Figure 1). Non-responders showed no change in LV or left atrial diameter (Figure 1).

After CRT, systolic LV function was markedly improved in responders, who showed a 52% increase in LVEF (12.5 increase in absolute percent units) (*p* < 0.001, Figure 2) and a 55% increase in fractional shortening (*p* < 0.05, Figure 2). LV systolic function was unchanged in the non-responders 3 months after CRT (Figure 2).

### Rho-Kinase Activation in PBMCs

**FIGURE 3:**
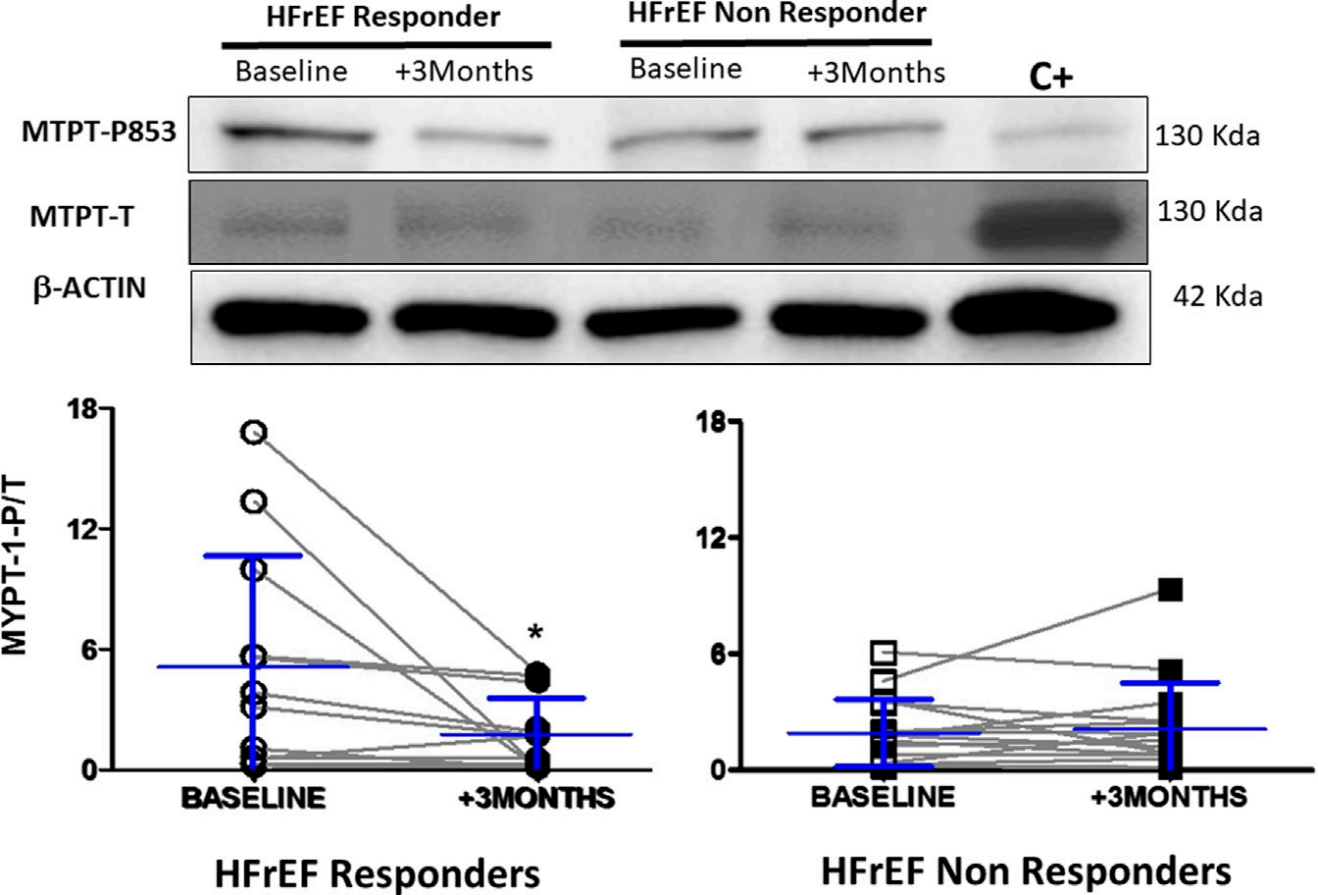
ROCK activity in circulating leukocytes measured by MYPT-1 phosphorylation in responders (*n* = 13) and non responders HFrEF patients (*n* = 15) before and after cardiac resynchronization therapy. Upper panel: Representative Western blots from one responder and one non responder HFrEF patient before and after cardiac resynchronization therapy. Lower panel: MYPT-1-p/t in both groups. Data shown as mean ± SD. Symbols: ** = *p*< 0.01 (non parametric Wilcoxon Signed Rank Test) vs baseline.

After CRT, ROCK activation was measured in PBMCs as MYPT1 phosphorylation (the relationship between phosphorylated and total MYPT1, or MYPT1-p/t). ROCK activation decreased significantly, by 3.3-fold, in responders HFrEF patients (*p* < 0.05, Figure 3) but was unchanged in non-responders (Figure 3). At baseline, no significant differences between responders and non-responders were observed.

### Apoptosis in Circulating Leukocytes of HFrEF Patients After CRT

**FIGURE 4:**
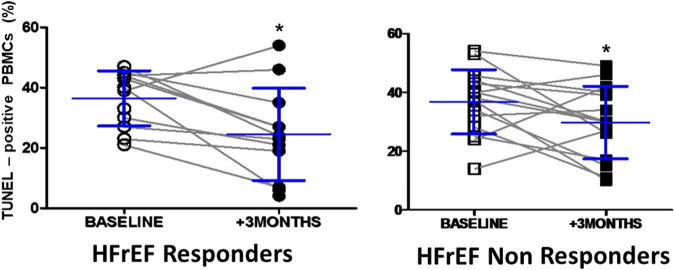
Apoptosis levels in circulating leukocytes in responders and non responders HFrEF patients before and after cardiac resynchronization therapy. TUNEL-positive nuclei baseline and 3 months after cardiac resynchronization therapy in responders and non responders HFrEF patients. Data shown as mean ± SD. Symbols: *p=<0.05 vs. baseline.

A significant and similar reduction in apoptosis levels after CRT was observed in PBMCs sampled from responders and non-responders (11.4 and 8% reductions, respectively, compared to baseline) (Figure 4).

## Discussion

The main finding of this proof-of-concept clinical study is that in HErEF patients, reverse cardiac remodeling 3 months after CRT is associated with a significant reduction in ROCK activation levels in PBMCs. This finding is consistent with the involvement of ROCK in myocardial remodeling in HFrEF. While small, this is the first clinical study to prospectively address the role of ROCK activation in reverse remodeling in human HFrEF. In a previous study, the ROCK inhibitor fasudil was administered for two weeks to patients with HF with preserved ejection fraction (HFpEF) and passive pulmonary hypertension. That study showed that fasudil improved pulmonary and left ventricular hemodynamics, suggesting that ROCK inhibition may be a promising target for HFpEF patients with a pulmonary hypertension phenotype (Zhang et al., 2018).

In the present study, HFrEF patients with effective CRT 3 months after device implantation (responders) showed reverse remodeling and reduced ROCK activation levels as well as significantly increased LVEF and reduced LV systolic, diastolic, and left atrial diameter. In contrast, the patients who did not show reverse remodeling 3 months after initiation of CRT (non-responders) also showed no change in ROCK activation levels. Interestingly, both groups (responders and non-responders) showed significant narrowing of the QRS complex (by 22.6 and 18.4%, respectively, NS), significantly increased distance on the 6 min walk test (107.7 and 75.4 m, respectively, NS), and significantly reduced TUNEL staining as a measure of apoptosis in PBMCs (by 11.4 and 8%, respectively, NS).

In stable HFrEF patients receiving optimal treatment, we have previously observed that ROCK pathway activation in PMBCs is related to adverse cardiac remodeling (Ocaranza et al., 2011), elevated catecholamines and Ang II levels, and low levels of Ang-(1–9), a vasodilatory angiotensin with cardioprotective effects (Ocaranza et al., 2020). The above finding that ROCK activation in PMBCs persists despite optimal treatment indicates that both neurohormonal activation and remodeling of the myocardium continue in these patients.

CRT enhances sarcomere contraction in cells by increasing calcium levels, enhancing cardiac contractility and systolic performance. Beta-adrenergic responses are increased after CRT by upregulation of the beta receptors on the myocardial cell surface. The above is important as cardiomyocytes in the failing heart generally have a reduced adrenergic response (downregulation) (Jaffe and Morin, 2014). In a preclinical model of HF with dyssynchrony, CRT reduced myocardial catecholamines to near control values (Chakir et al., 2009). Resynchronization therapy also increases adenosine triphosphate synthase activity by overturning an oxidative posttranslational modification in the mitochondria, leading to more effective energy metabolism and improved cardiomyocyte performance (Zweier et al., 2011).

In HFrEF patients with a wide QRS complex receiving resynchronization therapy, reverse remodeling is a rather consistent result (Linde et al., 2008; Moss et al., 2009; Jaffe and Morin, 2014). A recent study of 928 CRT patients followed for 3.8 years found that patients with ischemic cardiomyopathy achieve less reverse remodeling after CRT than patients with non-ischemic cardiomyopathy and that improved survival is closely related to the reverse remodeling observed (Kloosterman et al., 2020). This finding suggests that reverse remodeling should be evaluated and potentially used to predict outcome in clinical approaches to this disease (Kloosterman et al., 2020).

Reverse myocardial remodeling can be assessed in humans using magnetic resonance imaging, which can characterize the tissue and simultaneously assess the extent of scarring and dyssynchrony (Jaffe and Morin, 2014). In a clinical study of 68 patients with HFrEF and newly diagnosed DCM followed for 36 ± 24 months after hospital discharge, time-dependent modifications in myocardial characteristics were related to LV reverse remodeling (which was detected in 38% of the patients using cardiac magnetic resonance) (Nabeta et al., 2017). The authors observed that baseline late gadolinium enhancement and its evolution over time were both independently associated with subsequent LV reverse remodeling. Late gadolinium enhancement reflects both myocardial fibrosis (Abdel-Aty et al., 2005; Mewton et al., 2011; Yancy et al., 2013; Nabeta et al., 2014) and enlargement of the interstitial compartment as a consequence of myocardial pathologic remodeling, which involves inflammatory activity and edema (Vöringher et al., 2007; Mewton et al., 2011). In patients with severe aortic valve stenosis, reverse myocardial remodeling following surgery (aortic valve replacement) was assessed by magnetic resonance one year after surgery in 116 pacemaker-free survivors (Treibel et al., 2018). The authors found that diffuse fibrosis and myocardial hypertrophy recovered to more normal levels during the first 2 months after aortic valve replacement, whereas focal fibrosis was comparatively permanent (Treibel et al., 2018). All of these myocardial damage-related pathologic processes associated with reverse myocardial remodeling are promoted by ROCK activation (Shimizu and Liao, 2016; Dai et al., 2018; Yu et al., 2020). Our present findings of significant reverse remodeling 3 months after initiation of CRT in HFrEF are consistent with the role of ROCK activation in PBMCs as a marker of myocardial remodeling.

There are no studies in heart failure patients assessing ROCK activation levels and reverse remodeling. However, in 178 patients with congestive heart failure (mean LVEF 47.5%) followed for 2 years, the combination of ROCK activity in PBMCs and N-terminal pro–B-type natriuretic peptide (NT-proBNP) at baseline provided additional value in assessing long-term survival as compared to levels of this natriuretic peptide alone (Dong et al., 2012), suggesting that ROCK activation is associated with progressive cardiac remodeling and consequently with the pathogenesis of worsening HF (Do et al., 2013). Our current data comparing ROCK activation in HFrEF patients with and without reverse remodeling after CRT are consistent with this observation.

ROCK activation induces apoptosis in several tissues and cells (Mills et al., 1998; Coleman et al., 2001; Sebbagh et al., 2001), and cardiac apoptosis is a major mechanism of HF. Elevated apoptosis in circulating leukocytes has been observed in HFrEF patients compared to healthy controls, and these apoptosis levels correlate with ROCK activation in PBMCs (Ocaranza et al., 2020). Interestingly, in a preclinical model of ROCK activation secondary to genetically determined high angiotensin 1 converting enzyme and angiotensin II levels, increased ROCK-dependent apoptosis levels were found both in the LV and PBMCs (Ocaranza et al., 2020). These findings indicate that apoptosis in PBMCs parallels apoptosis in the myocardium. Here, TUNEL-positive PBMCs were comparably reduced 3 months after CRT in both groups, suggesting that in HFrEF patients, decreasing apoptosis in PBMCs contributes to, but is not specific of reverse myocardial remodeling.

We used PBMCs to assess the ROCK cardiac remodeling pathway in HFrEF patients for several reasons. First, PBMCs play a crucial role in the interface between the neuroendocrine and immune systems in response to stress. Second, these circulating cells share gene expression profiles with several tissues (Liew et al., 2006). Furthermore, two experimental studies that measured Rho-kinase phosphorylation simultaneously in the aorta, heart, and circulating leukocytes found a significant correlation between ROCK activation in circulating leukocytes and myocardial ROCK activity as well as aortic wall ROCK activity, which is in keeping with the idea that ROCK activation in PBMCs parallels ROCK activation in these two tissues (Fierro et al., 2016; Ocaranza et al., 2018). In addition, the convenience of circulating leukocytes for clinical examination as a noninvasive marker or target for serial observations is advantageous from a clinical perspective.

As CRT becomes more common, even among patients with wide QRS complexes, nearly 30% of patients do not achieve the desired clinical benefits (Birnie and Tang, 2006; Jaffe and Morin, 2014). We cannot rule out that a later assessment of LV size and function might show reverse remodeling and lower ROCK activation in PBMCs in the current non-responders. Other possible mechanisms potentially explaining non-response 3 months after CRT include: inconsistent pacing; lack of mechanical dyssynchrony, as no specific marker of LV dyssynchrony consistently predicts functional or clinical outcomes (Jaffe and Morin, 2014); scar burden in patients with ischemic cardiomyopathy; or lead position.

## Limitations

This clinical study has some limitations. The number of HFrEF patients is relatively small. However, the ROCK activation levels were significantly modified in patients with reverse remodeling but not in those without reverse remodeling was consistent within each group. Second, three months is a relatively short time to evaluate reverse remodeling after CRT in HFrEF patients. Nevertheless, the aim was to determine changes in ROCK activation levels before and after CRT in patients with and without reverse remodeling at the same timepoint. Our findings do not preclude the possibility that some non-responders could evolve to reverse remodeling at a later time.

## Conclusions

Effective CRT in HFrEF patients with reverse cardiac remodeling significantly decreases ROCK activity in PMBCs. Since ROCK activation has been shown to be a marker of cardiac remodeling and possibly a therapeutic target in these patients, reduced ROCK activation in PMBCs could be a marker of reverse cardiac remodeling. Further research is necessary to better assess this possibility.

## Data Availability

The raw data supporting the conclusions of this article will be made available by the authors, without undue reservation.

## References

[B1] Abdel-Aty H. Boyé P. Zagrosek A. Wassmuth R. Kumar A. Messroghli D. (2005). Diagnostic performance of cardiovascular magnetic resonance in patients with suspected acute myocarditis. J. Am. Coll. Cardiol. 45:1815–1822. 10.1016/j.jacc.2004.11.069 15936612

[B2] Abe S. Yoshihisa A. Ichijo Y. Sato Y. Kanno Y. Takiguchi M. (2020). Recovered left ventricular ejection fraction and its prognostic impacts in hospitalized heart failure patients with reduced ejection fraction. Int. Heart J. 61:281. 10.1536/ihj.19-211 31956135

[B3] Birnie D. H. Tang A. S. (2006). The problem of non-response to cardiac resynchronization therapy. Curr. Opin. Cardiol. 21:20–26. 10.1097/01.hco.0000198983.93755.99 16355025

[B4] Chakir K. Daya S. K. Aiba T. Tunin R. S. Dimaano V. L. Abraham T. P. (2009). Mechanisms of enhanced β-adrenergic reserve from cardiac resynchronization therapy. Circulation 119:1231–1240. 10.1161/CIRCULATIONAHA.108.774752 19237665 PMC2850078

[B5] Choi J.-O. Kim E. Y. Lee G. Y. Lee S.-C. Park S. W. Kim D.-K. (2013). Predictors of left ventricular reverse remodeling and subsequent outcome in nonischemic dilated cardiomyopathy. Circ. J. 77: 462–469. 10.1253/circj.cj-12-0507 23095684

[B6] Coleman M. L. Sahai E. A. Yeo M. Bosch M. Dewar A. Olson M. F. (2001). Membrane blebbing during apoptosis results from caspase-mediated activation of ROCK I. Nat. Cel Biol. 3:339–345. 10.1038/35070009 11283606

[B7] Dai Y. Luo W. Chang J. (2018). Rho kinase signaling and cardiac physiology. Curr. Opin. Physiol. 1:14. 10.1016/j.cophys.2017.07.005 29527586 PMC5842951

[B8] Do e Z. Fukumoto Y. Sugimura K. Miura Y. Tatebe S. Yamamoto S. (2013). Rho-kinase activation in patients with heart failure. Circ. J. 77:2542–2550. 10.1253/circj.cj-13-0397 23883874

[B9] Dong M. Liao J. K. Fang F. Lee A. P.-W. Yan B. P.-Y. Liu M. (2012). Increased Rho kinase activity in congestive heart failure. Eur. J. Heart Fail. 14:965–973. 10.1093/eurjhf/hfs068 22588320 PMC3707433

[B10] Epstein A. E. DiMarco J. P. Ellenbogen K. A. Estes N. A. Freedman R. A. Gettes L. S. (2013). 2012 ACCF/AHA/HRS focused update incorporated into the ACCF/AHA/HRS 2008 guidelines for device-based therapy of cardiac rhythm abnormalities: a report of the American College of Cardiology foundation/American heart association task force on practice guidelines and the heart rhythm society. Circulation 127:e283-352. 10.1016/j.jacc.2012.11.007 23255456

[B11] Fierro C. Novoa U. González V. Ocaranza M. P. Jalil J. E. (2016). Simultaneous Rho kinase inhibition in circulating leukocytes and in cardiovascular tissue in rats with high angiotensin converting enzyme levels. Int. J. Cardiol. 215:309–317. 10.1016/j.ijcard.2016.04.004 27128553

[B12] Gabrielli L. Winter J. L. Godoy I. McNab P. Padilla I. Cordova S. (2014). Increased rho-kinase activity in hypertensive patients with left ventricular hypertrophy. Am. J. Hypertens. 27:838–845. 10.1093/ajh/hpt234 24363278

[B13] Hartmann S. Ridley A. J. Lutz S. (2015). The function of rho-associated kinases ROCK1 and ROCK2 in the pathogenesis of cardiovascular disease. Front. Pharmacol. 6:276. 10.3389/fphar.2015.00276 26635606 PMC4653301

[B14] Hsu J. C. Solomon S. D. Bourgoun M. McNitt S. Goldenberg I. Klein H. (2012). Predictors of super-response to cardiac resynchronization therapy and associated improvement in clinical outcome. J. Am. Coll. Cardiol. 59: 2366–2373. 10.1016/j.jacc.2012.01.065 22698490

[B15] Jaffe L. M. Morin D. P. (2014). Cardiac resynchronization therapy: history, present status, and future directions. Ochsner J. 14:596–507. PMID: 25598725; PMCID: PMC4295737. 25598725 PMC4295737

[B16] Jalil J. Lavandero S. Chiong M. Paz Ocaranza M. (2005). Rho/Rho kinase signal transduction pathway in cardiovascular disease and cardiovascular remodeling. Revista Española de Cardiología (English Edition) 58:951–961. 10.1016/S1885-5857(06)60378-2 16053829

[B17] Kloosterman M. Stipdonk A. M. W. Horst I. Rienstra M. Van Gelder I. C. Vos M. A. (2020). Association between heart failure aetiology and magnitude of echocardiographic remodelling and outcome of cardiac resynchronization therapy. ESC Heart Fail. 7:645. 10.1002/ehf2.12624 31991067 PMC7160473

[B18] Kobayashi N. Horinaka S. Mita S. Nakano S. Honda T. Yoshida K. (2002). Critical role of Rho-kinase pathway for cardiac performance and remodeling in failing rat hearts. Cardiovasc. Res. 55:757–767. 10.1016/s0008-6363(02)00457-1 12176125

[B19] Lang R. M. Bierig M. Devereux R. B. Flachskampf F. A. Foster E. Pellikka P. A. (2005) Recommendations for chamber quantification: a report from the American society of echocardiography's guidelines and standards committee and the chamber quantification writing group, developed in conjunction with the European association of echocardiography, a branch of the European society of Cardiology. J. Am. Soc. Echocardiography 18:1440–1463. 10.1016/j.echo.2005.10.005 16376782

[B20] Liew C.-C. Ma J. Tang H.-C. Zheng R. Dempsey A. A. (2006). The peripheral blood transcriptome dynamically reflects system wide biology: a potential diagnostic tool. J. Lab. Clin. Med. 147:126–132. 10.1016/j.lab.2005.10.005 16503242

[B21] Linde C. Abraham W. T. Gold M. R. St. John Sutton M. Ghio S. Daubert C. (2008). Randomized trial of cardiac resynchronization in mildly symptomatic heart failure patients and in asymptomatic patients with left ventricular dysfunction and previous heart failure symptoms. J. Am. Coll. Cardiol. 52:1834–1843. 10.1016/j.jacc.2008.08.027 19038680

[B22] Loirand G. Pacaud P. (2014). Involvement of Rho GTPases and their regulators in the pathogenesis of hypertension. Small GTPases 5:e983866-10. 10.4161/sgtp.28846 PMC4205133 25496262

[B23] Maki H. Takeda N. (2020). Reverse remodeling and current medical therapy in heart failure with reduced ejection fraction. Int. Heart J. 61: 197–198. 10.1536/ihj.20-034 32224600

[B24] Mera C. Godoy I. Ramírez R. Moya J. Ocaranza M. P. Jalil J. E. (2016). Mechanisms of favorable effects of Rho kinase inhibition on myocardial remodeling and systolic function after experimental myocardial infarction in the rat. Ther. Adv. Cardiovasc. Dis. 10:4–20. 10.1177/1753944715609516 26490279 PMC5933601

[B25] Mewton N. Liu C. Y. Croisille P. Bluemke D. Lima J. A. C. (2011). Assessment of myocardial fibrosis with cardiovascular magnetic resonance. J. Am. Coll. Cardiol. 57:891. 10.1016/j.jacc.2010.11.013 21329834 PMC3081658

[B26] Mills J. C. Stone N. L. Erhardt J. Pittman R. N. (1998). Apoptotic membrane blebbing is regulated by myosin light chain phosphorylation. J. Cel Biol. 140:627–636. 10.1083/jcb.140.3.627 PMC2140178 9456322

[B27] Moss A. J. Hall W. J. Cannom D. S. Klein H. Brown M. W. Daubert J. P. (2009). Cardiac-resynchronization therapy for the prevention of heart-failure events. N. Engl. J. Med. 361:1329- 1338. 10.1056/NEJMoa0906431 19723701

[B28] Nabeta T. Inomata T. Fujita T. Iida Y. Ikeda Y. Sato T. (2017). Temporal change of myocardial tissue character is associated with left ventricular reverse remodeling in patients with dilated cardiomyopathy: a cardiovascular magnetic resonance study. J. Cardiol. 70:185. 10.1016/j.jjcc.2016.10.017 27979675

[B29] Nabeta T. Inomata T. Iida Y. Ikeda Y. Iwamoto M. Ishii S. (2014). Baseline cardiac magnetic resonance imaging versus baseline endomyocardial biopsy for the prediction of left ventricular reverse remodeling and prognosis in response to therapy in patients with idiopathic dilated cardiomyopathy. Heart Vessels 29:784–792. 10.1007/s00380-013-0415-1 24092362 PMC4226927

[B30] Ocaranza M. P. Fierro C. Jalil J. E. Moya J. Gonzalez L. Molina C. (2018). Rho kinase activation in circulating leukocytes is related to hypertensive myocardial remodeling. Clin. Sci. (Lond). 132:1837–1853. 10.1042/CS20180312 30065083

[B31] Ocaranza M. P. Gabrielli L. Mora I. Garcia L. McNab P. Godoy I. (2011). Markedly increased Rho-kinase activity in circulating leukocytes in patients with chronic heart failure. Am. Heart J. 161:931–937. 10.1016/j.ahj.2011.01.024 21570525

[B32] Ocaranza M. P. Moya J. Jalil J. E. Lavandero S. Kalergis A. M. Molina C. (2020). Rho‐kinase pathway activation and apoptosis in circulating leucocytes in patients with heart failure with reduced ejection fraction. J. Cel Mol Med. 24:1413–1427. 10.111110.1111/jcmm.14819 PMC6991691 31778027

[B33] Reichart D. Magnussen C. Zeller T. Blankenberg S. (2019). Dilated cardiomyopathy: from epidemiologic to genetic phenotypes. J. Intern. Med. 286:362. 10.1111/joim.12944 31132311

[B34] Ruwald M. H. Solomon S. D. Foster E. Kutyifa V. Ruwald A.-C. Sherazi S. (2014). Left ventricular ejection fraction normalization in cardiac resynchronization therapy and risk of ventricular arrhythmias and clinical outcomes. Circulation 130: 2278–2286. 10.1161/CIRCULATIONAHA.114.011283 25301831

[B35] Sebbagh M. Renvoizé C. Hamelin J. Riché N. Bertoglio J. Bréard J. (2001). Caspase-3-mediated cleavage of ROCK I induces MLC phosphorylation and apoptotic membrane blebbing. Nat. Cel Biol. 3:346–352. 10.1038/35070019 11283607

[B36] Shimizu T. Liao J. K. (2016). Rho kinases and cardiac remodeling. Circ. J. 80:1491. 10.1253/circj.CJ-16-0433 27251065 PMC5563468

[B37] Shimokawa H. Sunamura S. Satoh K. (2016). RhoA/rho-kinase in the cardiovascular system. Circ. Res. 118:352–366. 10.1161/CIRCRESAHA.115.306532 26838319

[B38] Surma M. Wei L. Shi J. (2011). Rho kinase as a therapeutic target in cardiovascular disease. Future Cardiol. 7:657–671. 10.2217/fca.11.51 21929346 PMC3193795

[B39] Sze E. Daubert J. P. (2018). Left bundle branch block-induced left ventricular remodeling and its potential for reverse remodeling. J. Interv. Card. Electrophysiol. 52:343–352. 10.1007/s10840-018-0407-2 30019271

[B40] Tardif J.-C. O'Meara E. Komajda M. Böhm M. Borer J. S. Ford I. (2011). Effects of selective heart rate reduction with ivabradine on left ventricular remodelling and function: results from the SHIFT echocardiography substudy. Eur. Heart J. 32: 2507–2515. 10.1093/eurheartj/ehr311 21875858 PMC3195263

[B41] Treibel T. A. Kozor R. Schofield R. Benedetti G. Fontana M. Bhuva A. N. (2018). Reverse myocardial remodeling following valve replacement in patients with aortic stenosis. J. Am. Coll. Cardiol. 71:860–871. 10.1016/j.jacc.2017.12.035 29471937 PMC5821681

[B42] Vöhringer M. Mahrholdt H. Yilmaz A. Sechtem U. (2007). Significance of late gadolinium enhancement in cardiovascular magnetic resonance imaging (CMR). Herz 32:129. 10.1007/s00059-007-2972-5 17401755

[B43] Yancy C. W. Jessup M. Bozkurt B. Butler J. Casey D. E. Jr. Drazner M. H. (2013). 2013 ACCF/AHA guideline for the management of heart failure: executive summary. Circulation 128:1810–1852. 10.1161/CIR.0b013e31829e8807 23741057

[B44] Yu B. Sladojevic N. Blair J. E. Liao J. K. (2020). Targeting Rho-associated coiled-coil forming protein kinase (ROCK) in cardiovascular fibrosis and stiffening. Expert Opin. Ther. Targets 24:47. 10.1080/14728222.2020.1712593 31906742 PMC7662835

[B45] Zhang X. Zhang X. Wang S. Luo J. Zhao Z. Zheng C. (2018). Effects of fasudil on patients with pulmonary hypertension associated with left ventricular heart failure with preserved ejection fraction: a prospective intervention study. Can. Respir. J. 2018. 1. 10.1155/2018/3148259 PMC5892244 29785232

[B46] Zweier J. L. Chen C.-A. Talukder M. A. H. (2011). Cardiac resynchronization therapy and reverse molecular remodeling. Circ. Res. 109:716–719. 10.1161/CIRCRESAHA.111.253864 21921269 PMC4073596

